# Extra-Ocular Sebaceous Carcinoma Unveiled: A Case Study of Dermal Challenges in an Elderly African American Patient

**DOI:** 10.7759/cureus.51934

**Published:** 2024-01-09

**Authors:** Kaitlyn Pearl, Patricia Zielinski, Bernard J Zaragoza

**Affiliations:** 1 Dr. Kiran C. Patel College of Osteopathic Medicine, Nova Southeastern University, Fort Lauderdale, USA; 2 Department of Surgery, Broward Health Coral Springs, Coral Springs, USA

**Keywords:** sebaceous carcinoma, malignancy surgery, derm path, cutaneous oncology, extraocular sebaceous carcinoma, back mass, dermatology

## Abstract

Sebaceous carcinoma is a rare malignant tumor of the sebaceous glands that most commonly presents in the periocular area. Uncommon extraocular manifestations are occasionally observed, often limited to the head or neck but can occur wherever sebaceous glands are found. There are increasing cases of sebaceous carcinoma in the United States which continue to pose ongoing diagnostic challenges. We present a patient with a 5.5 x 4 x 2 cm gradually growing necrotic and fungating upper back mass, present for one year. This lesion was initially diagnosed as an excoriated sebaceous adenoma, microscopically showing well-formed organoid structures and no irregular infiltration into the dermis, but was later confirmed as sebaceous carcinoma, demonstrating prominent mitosis and infiltrative growth pattern, following wide surgical excision. Margins were clear following the surgery with no signs of recurrence but further treatment recommendations included follow-up with oncology due to the rarity and aggressive nature of this tumor. This case serves to present an atypical presentation of a rare malignancy that has an increased occurrence rate for unknown reasons.

## Introduction

Sebaceous carcinoma is a rare malignant tumor of the sebaceous glands. While it most commonly presents in the periocular area, head, or neck, it can arise anywhere sebaceous glands are located. Most frequently in the periocular region, it affects the modified sebaceous glands and Meibomian glands, with more than 34.5% of cases occurring on the upper eyelid due to the higher concentration of Meibomian glands in this area [1,2]. Extraocular presentations are less common with 70% of reported cases located in the head/neck region and only around 13% manifesting on the back or trunk [2,3]. 

The incidence of sebaceous carcinoma has been increasing in the United States; however, the underlying reasons for this change are unclear [3]. Sebaceous carcinoma exhibits a higher incidence in men, 0.32 per 100,000 person-years, than in women, 0.16 per 100,000 person-years [3]. The overall prevalence of this condition is estimated at one to two cases per 1,000,000 person-years with an expected five-year overall survival rate of 78% for localized/regional disease and 50% for cases with metastatic spread [3,4]. In 98% of cases, sebaceous carcinoma has occurred in individuals aged above 40 years, with most occurring in the seventh and eighth decade of life, and non-Hispanic Caucasians have a four-fold increased incidence compared with African Americans [1,3].

In this report, we describe a case involving a progressively enlarging upper back mass. The initial diagnosis suggested a sebaceous adenoma; however, subsequent surgical excision confirmed the presence of sebaceous carcinoma. This case underscores the complexities and diagnostic challenges given the rarity of this pathology.

## Case presentation

An 80-year-old man of African American descent presented to his primary care doctor with a large pedunculated, fungating, and ulcerated mass located on his right upper back with an initial presentation of one year. The mass exhibited gradual enlargement over time, and the patient did not experience any pain; instead, he reported discomfort when the mass was impacted or jarred. The patient also noted experiencing constant itchiness in the area but did not report any bleeding episodes. He is a former smoker with a past medical history significant for benign essential hypertension, severe chronic obstructive pulmonary disease (COPD), Stage 3a chronic kidney disease, mixed hyperlipidemia, peripheral vascular disease, peripheral neuropathy, low tension glaucoma, elevated prostate-specific antigen (PSA), and primary non-thrombocytopenic purpura. The patient reported the absence of any pertinent family history. At presentation, he did not meet any of the predisposing risk factors associated with sebaceous carcinoma such as immunosuppression, Muir-Torre syndrome (MTS), or a personal or family history of internal malignancies such as colorectal and genitourinary cancers. 

**Figure 1:**
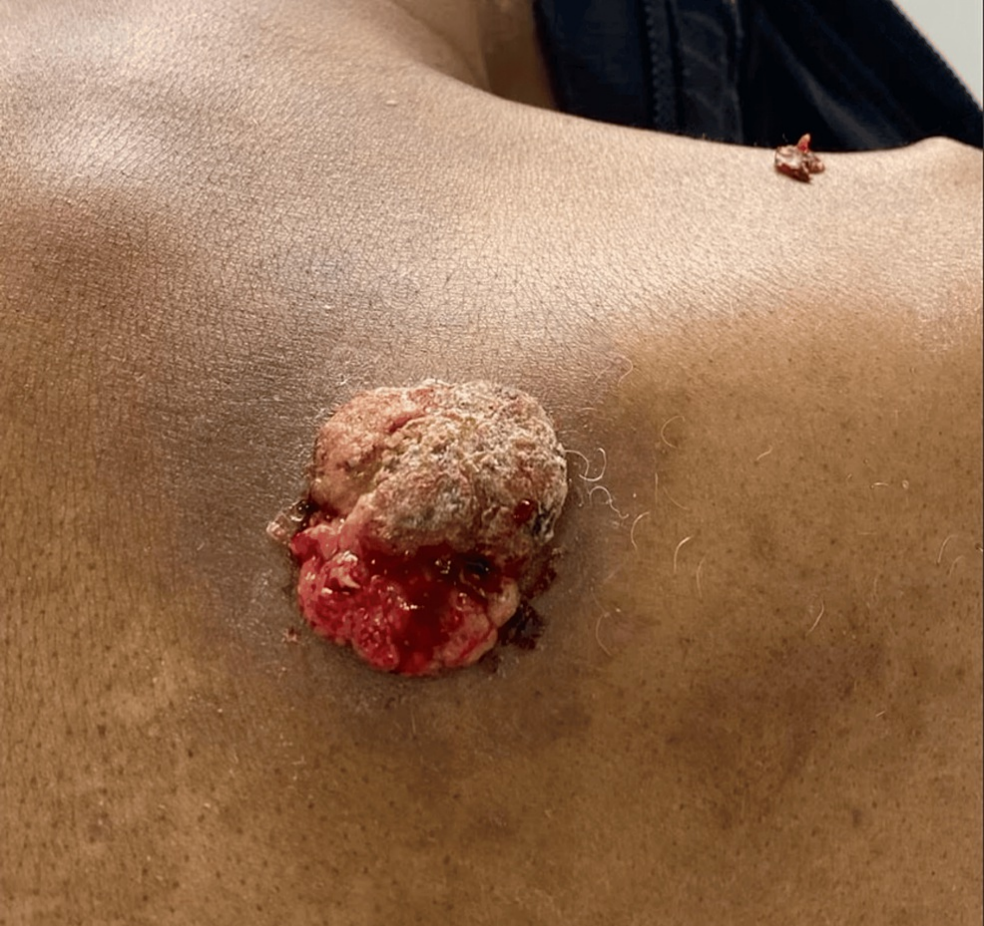
Pre-operative sebaceous neoplasm on right upper back taken at time of initial biopsy.

On physical examination, there was a 5.5 x 4 x 2 cm necrotic fungating mass (Figure 1). No swelling in regional lymph nodes or signs of metastasis were noted. The lesion was found to be a solitary nodule and skin biopsy was indicated. The original skin biopsy of the lesion, completed on March 1, 2023, was consistent with excoriated sebaceous adenoma. The gross sample measured 1.0 x 0.8 x 0.2 cm and microscopically showed well-formed organoid structures that showed several layers of germinative cells maturing into sebocytes as well as differentiating into duct-like structures. There was no irregular infiltration into the dermis that was evident and germinative cells did not outnumber sebocytes. Due to the anticipated benign nature of this condition, no additional diagnostic testing was deemed necessary. The recommendation was for the patient to pursue outpatient elective surgery for removal of the mass.

**Figure 2:**
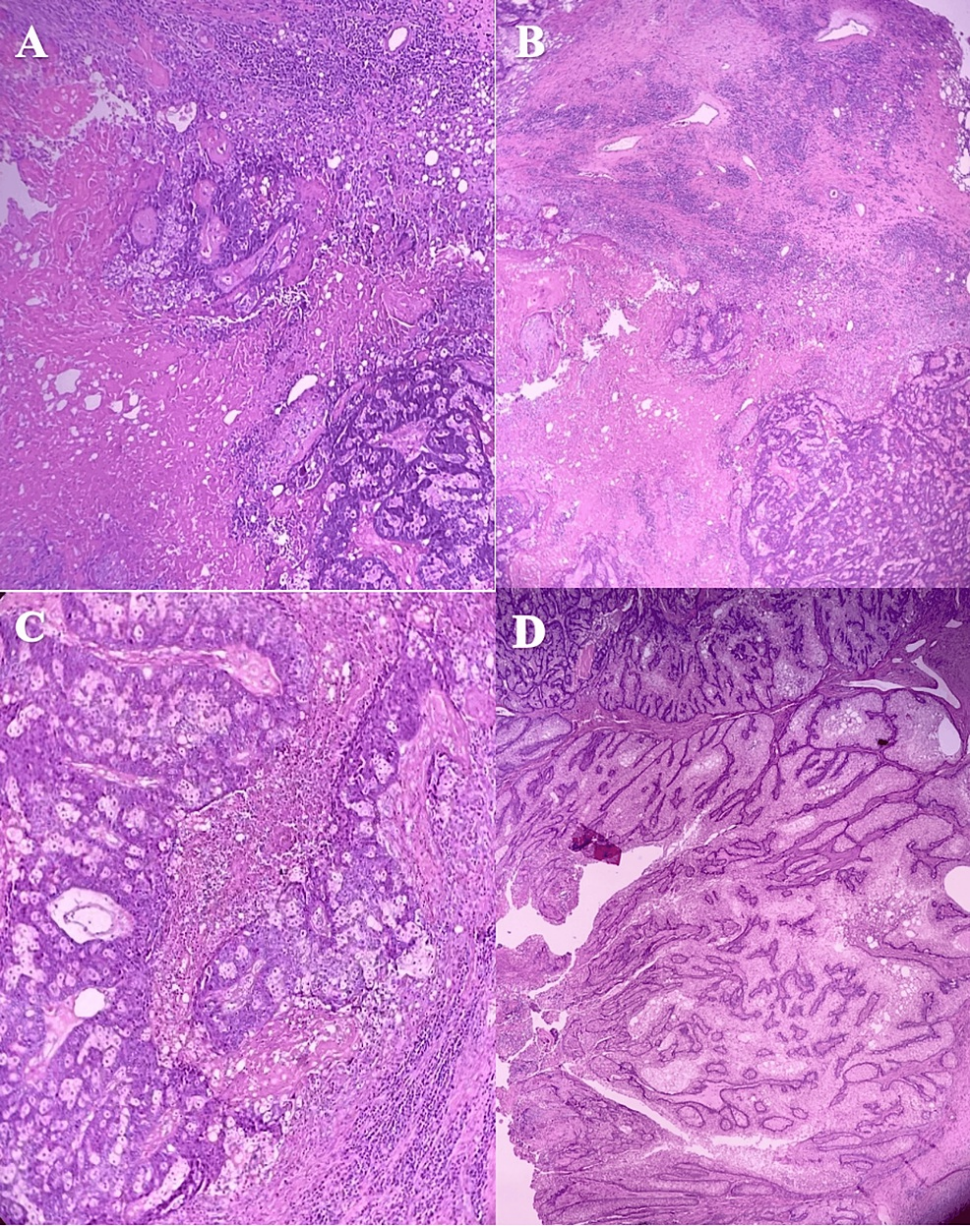
Histologic evaluation consistent with sebaceous carcinoma, H&E. (A) Infiltrative growth pattern with a central area of necrosis; (B) Malignant cells with numerous mitotic figures and vacuolated cells with centrally located nuclei; (C) Sebaceous carcinoma with pleomorphism and scattered mitosis; (D) Well-differentiated portion of sebaceous carcinoma closely resembling sebaceous adenoma.

Following the continued growth of the mass, the patient presented for surgical intervention and mass removal. During surgery, the mass was entirely excised with 5 mm margins circumferentially using an elliptical incision and dissection through the subcutaneous tissue. The skin was then closed using interrupted 4.0 Monocryl (Ethicon, Inc., Raritan, New Jersey, United States) in a subcuticular fashion. No pre or post-operative medications were prescribed. The final pathology report on September 9, 2023, showed a well-differentiated sebaceous carcinoma with negative margins of resection. Grossly, the lesion was described as a right upper back mass with a wrinkled, keratotic brown-gray skin ellipse measuring 3.5 x 2 cm excised to a depth of 0.8 cm. The epidermis displayed a 5.5 x 4 x 2 cm necrotic, fungating nodule located 0.7 cm from the nearest (3 o’clock) margin. Sectioning revealed tan-yellow and orange cut surfaces. Microscopically there was a prominent mitosis and infiltrative growth pattern (Figure 2). Due to its rarity, no specific staging criteria for extraocular sebaceous carcinoma exist [5].

**Figure 3:**
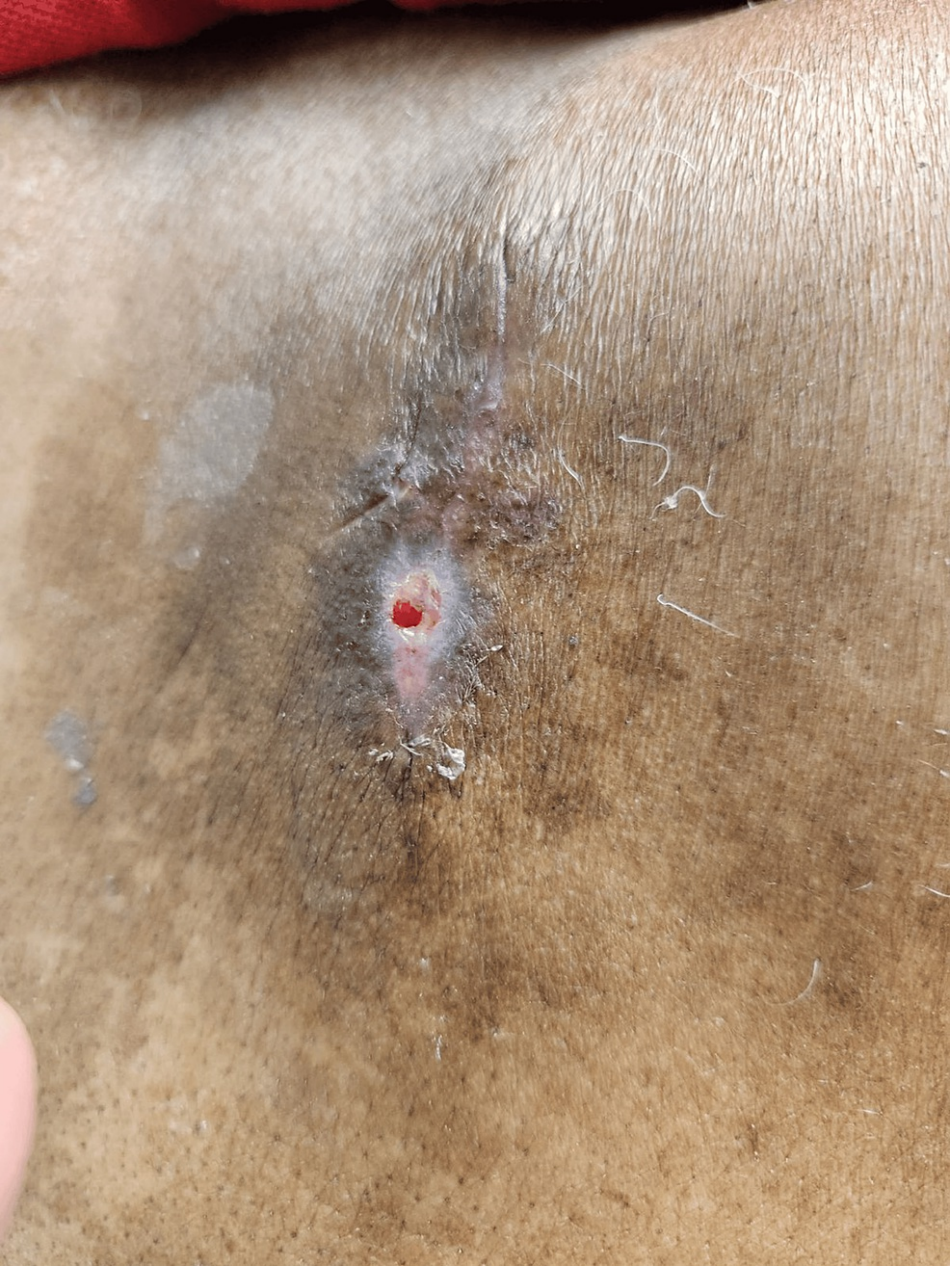
Three-week post-operative image of excision site on patient’s upper back.

After the post-operative pathology results, the patient was instructed to follow up with oncology. Three weeks post-operatively, the site was healing well and there was no sign of recurrence (Figure 3). At the six-week post-operative follow-up, he reported doing well and had no complaints following the excision and declined further follow-up with an oncologist at this time.

## Discussion

Sebaceous carcinoma is a rare skin cancer that has been diagnosed in a wide array of individuals. It most commonly appears in patients aged 60-80 with non-Hispanic White individuals comprising 78% of cases [2,3]. Notably, the current case presentation gains added significance since associations with male sex, Black race, and extraocular anatomic locations are associated with a significantly higher all-cause mortality [2]. Most sebaceous carcinomas occur from de novo mutations, but they can also arise from a previous benign sebaceous tumor. However, this is much less common [1], further complicating the initial management of our patient.

The occurrence of sebaceous carcinoma is often associated with recurring growth, local infiltration, and the potential for distant metastasis via lymphatic or hematogenous dissemination, which are prevalent complications [1]. Some cases are associated with MTS, a form of Lynch syndrome, with a loss of mismatch repair genes and microsatellite instability. In MTS, sebaceous tumors can occur concurrently with other internal malignancies, most commonly colorectal and genitourinary cancers. Lastly, malignancies in MTS may occur spontaneously without a loss of mismatch repair genes [1]. Risks for sebaceous carcinoma further increase with exposure to ultraviolet (UV) radiation, immunosuppression, MTS/genetic susceptibility, and increasing age [2]. As his primary residence was in Florida, potential heightened UV radiation exposure and older age could have increased the likelihood of the patient developing a sebaceous carcinoma [6]. Due to a lack of personal or pertinent family history, screening for MTS was not indicated at this time. 

When sebaceous carcinoma presents extra-ocularly, it usually presents as an ulcerated yellowish nodule. Aside from presenting on the head and neck, there have been reported cases also on the parotid gland, prostate, breast, and ovary [1]. Histopathologically it is divided into four categories: papillary, nodular, comedocarcinoma, and mixed, and can range from poor to well-differentiated. It is defined by irregular asymmetric sebaceous lobules within the dermis with malignant cells having substantial nuclear atypia and mitotic activity, hyperchromatism, and pleomorphism [1]. The final histopathology in our case shows these features of necrosis, pleomorphism, scattered mitosis, and well-differentiated carcinoma that closely resembles sebaceous adenoma. 

A case similar to the one we are presenting is of a carcinoma that arose from a single nodule of benign sebaceoma located on the posterior neck in a 74-year-old Asian woman present for one year [7]. Analogous to the current case, this one was also initially biopsied as a benign lesion, a sebaceoma; however, the lesion had a combination of both benign and malignant components. Therefore, a deep biopsy may be required for accurate diagnosis, as a superficial sample may only encompass the typical histopathological features of a benign sebaceoma or adenoma, and exclude the malignant aspects [7]. Whether or not this is what occurred in the initial biopsy of the patient in our case, this point should be emphasized as a delay in diagnosis can be detrimental due to the aggressive nature of this malignancy. 

The pathogenesis of sebaceous carcinoma is not well understood but is multifactorial with dysregulation in the NF-κB (nuclear factor kappa B), PTEN (Phosphatase and TENsin homolog deleted on chromosome 10), and TGF-β (transforming growth factor-β) signaling pathways when comparing sebaceous carcinoma to adenoma [8]. In addition, there is a difference in the predominance of genes in ocular vs. extraocular sebaceous carcinomas, with *TP53* and *PIK3CA* being more frequently mutated in ocular and *NOTCH1* gene more commonly mutated in extraocular sebaceous carcinomas [9]. 

It is important to distinguish sebaceous carcinoma from other more common tumors that can present similarly. For instance, an inflamed or irritated chalazion, papilloma, keratoacanthoma, and inclusion or dermoid cyst can all be included in the differential of an eyelid tumor [1]. Importantly, a nonmalignant tumor that may mimic sebaceous carcinoma, a sebaceous adenoma, does not commonly occur in the periocular region [1]. Basal cell carcinoma, squamous cell carcinoma, and Merkel cell carcinoma are other malignancies that must be differentiated from sebaceous carcinoma. Basal cell and squamous cell carcinoma are the most common type of cancer in the United States, much more common than sebaceous carcinoma, with research estimating over 5.4 million basal and squamous cell carcinomas are diagnosed in over three million Americans per year [10]. Even though these are the most common types of cancer, mortality rates are very low with 2,000-8,000 deaths per year, mostly from squamous cell carcinoma [10]. Since clinical presentation can appear similarly among different pathologies, biopsies are essential to diagnose sebaceous carcinoma and differentiate it from more common types of lesions. Hematoxylin and eosin stain is routine to confirm the diagnosis, as in the case present in this report, but immunohistochemical staining may also play a role. Sebaceous carcinomas display nearly 100% epithelial membrane antigen, adipophilin, and androgen receptor positivity [1]. 

Sebaceous carcinomas are considered aggressive due to metastatic potential with extraocular manifestations having poorer five-year survival rates (68%) compared to those with ocular malignancies (75.2%) [1]. Spread to the lungs, liver, brain, small intestine, and urinary tract have been reported, and metastasis decreases the five-year survival rate to 50% [1,2]. In extraocular malignancies, such as the one reported in this case, the recurrence rate is 29% while the metastasis rate is 21% [1]. Since these tumors are most commonly present on the head and neck, early identification and treatment are crucial to decrease complex oncologic management and facial reconstruction. 

Given its uncommon presentation, there is a lack of well-established guidelines for the management of sebaceous carcinoma. First-line treatment of sebaceous carcinoma includes surgical excision with the use of wide margins and frozen sections or Mohs micrographic surgery [1,3]. After an initial biopsy at the patient's primary care physician's office, suspicion of malignancy arose due to ongoing growth and the anticipated infiltrative growth pattern, which was confirmed through histological diagnosis following excision. Although 5 mm margins were employed and verified as negative during excision, the specific application of margin sizes to prevent recurrence remains a subject with limited consensus in the medical literature. Generally, 5-6 mm margins or up to 1 cm are considered standard [11]. Also, there are no distinct guidelines concerning systemic evaluation; however, those with large tumors or longstanding tumors may benefit from imaging with CT or MRI to check for metastasis. Those with MTS may also undergo a positron emission tomography (PET) scan to look for any malignancy that has spread to the viscera [1]. In the current case, the patient was referred to oncology for management and examination to rule out possible distant metastasis. The present case supplements current literature and notable case presentations on the management and treatment of sebaceous carcinoma and can be referenced when evaluating atypical presentation and methodology in surgical interventions.

## Conclusions

The growing incidence of sebaceous carcinoma poses an ongoing challenge, underscoring the critical need for accurate identification and treatment to improve survivability, particularly in atypical presentations. This case study highlights the diagnostic intricacies and potential for misdiagnosis associated with sebaceous carcinoma, especially in distinguishing between incisional and excisional biopsy results. There is an escalating need for further scientific research to explore risk factors and associations, given the rising incidence of this malignancy. The presented case contributes valuable insights to the current medical literature, enriching our understanding of the optimal management and treatment strategies for these tumors. In instances of extraocular presentations, as exemplified in the present case, sebaceous carcinoma should not be excluded from the differential diagnosis. Its shared characteristics with more prevalent pathologies and benign adenomas emphasize the importance of exercising caution to prevent delays in diagnosing this rare yet highly aggressive tumor.
